# Trans-Reflective Color Filters Based on a Phase Compensated Etalon Enabling Adjustable Color Saturation

**DOI:** 10.1038/srep25496

**Published:** 2016-05-06

**Authors:** Chul-Soon Park, Vivek Raj Shrestha, Sang-Shin Lee, Duk-Yong Choi

**Affiliations:** 1 Department of Electronic Engineering, Kwangwoon University, 20 Kwangwoon-ro, Nowon-gu, Seoul 01897, South Korea; 2 Laser Physics Centre, Research School of Physics and Engineering, Australian National University, Canberra ACT 2601, Australia

## Abstract

Trans-reflective color filters, which take advantage of a phase compensated etalon (silver-titania-silver-titania) based nano-resonator, have been demonstrated to feature a variable spectral bandwidth at a constant resonant wavelength. Such adjustment of the bandwidth is presumed to translate into flexible control of the color saturation for the transmissive and reflective output colors produced by the filters. The thickness of the metallic mirror is primarily altered to tailor the bandwidth, which however entails a phase shift associated with the etalon. As a result, the resonant wavelength is inevitably displaced. In order to mitigate this issue, we attempted to compensate for the induced phase shift by introducing a dielectric functional layer on top of the etalon. The phase compensation mediated by the functional layer was meticulously investigated in terms of the thickness of the metallic mirror, from the perspective of the resonance condition. The proposed color filters were capable of providing additive colors of blue, green, and red for the transmission mode while exhibiting subtractive colors of yellow, magenta, and cyan for the reflection mode. The corresponding color saturation was estimated to be efficiently adjusted both in transmission and reflection.

Nano-structured color filters have gained a great deal of interest as a pivotal element in a variety of applications for display/imaging, sensing, color printing, and photovoltaics^1^,^2^. The nano-structured color filters can realize a broad color palette by mimicking micro/nano structures in nature, such as that of a Morpho butterfly’s wings^3^,^4^,^5^,^6^. Unlike conventional colorant pigment based filters, such structural filters are believed to be impervious to heat exposure, ultra-violet irradiation, and moisture^7^,^8^,^9^,^10^. To date, diverse structural colors have been reported based on plasmonic devices^9^,^10^,^11^,^12^,^13^,^14^,^15^,^16^, guided-mode resonance filters^17^,^18^,^19^,^20^,^21^, and etalon resonators of metal-dielectric-metal (MDM) configuration^22^,^23^,^24^,^25^,^26^,^27^,^28^,^29^. In particular, due to its salient features of simple structure and angular tolerance^23^,^24^,^25^,^26^,^27^, the etalon nano-resonator has been extensively adopted for embodying color filters. It is noted that silver (Ag) plays an integral role as a metallic mirror, in light of its low extinction coefficient and inter-band transitions that cause an optical loss in the visible band^30^. Transmissive and reflective color filters have previously been attempted by using relatively thin and thick Ag mirror layers, respectively^22^,^23^,^24^,^25^,^26^. Reflective devices employing a perfect absorber have also been reported^27^,^28^,^29^. However, it is accepted that a trans-reflective color filter is categorically integral to such applications as the holography, fluorescence microscopy, CCD imaging, and so forth^31^,^32^. In order to efficiently manipulate the color saturation of its output, such a trans-reflective color filter is strongly preferred to allow a flexibly tailored bandwidth while preserving the resonant wavelength^21^.

In this paper, we present trans-reflective color filters, while taking advantage of a phase compensated etalon (PCE) based nano-resonator, allowing for an adjustable bandwidth at a constant resonant wavelength. Each filter consists of a silver-titania-silver (Ag-TiO_2_-Ag) structure integrated with a dielectric functional layer (DFL) in titania, thus assuming a (metal-dielectric-metal)|dielectric (MDMD) configuration. The proposed filter was rigorously inspected in terms of the resonant wavelength in accordance with the metal thickness. The DFL was validated to play a crucial role in adequately compensating for the phase shift incurred depending on the thickness of the metallic mirror, thereby preserving the resonant wavelength. In our previous work^24^, for a set of etalon color filters a phase compensation scheme based on a dielectric overlay was proposed and investigated to mainly improve their efficiency and angular sensitivity. However, the performance seriously hinges on the thickness of metallic mirrors associated with the etalon, which is responsible for the total accumulated phase shift within the cavity. In an attempt to mitigate that issue, in this work we endeavored to newly develop highly efficient trans-reflective color filters, enabling substantially relaxed tolerance in terms of the thickness of metallic mirrors, which would be of tremendous importance in fabrication. Another remarkable aspect is relevant to flexible control and substantial enhancement of the color saturation of the filter devices. From the viewpoint of practical applications, the color saturation, an attribute of visual perception, should be preferably adjusted while the resonant wavelength is stably retained in a certain color domain. The color saturation is principally determined by the spectral bandwidth^21^, which can be tailored by the thickness of metallic mirrors. However, the resonant wavelength might be inevitably altered by the total phase shift depending on the mirror thickness, so that the dominant wavelength is displaced so as to ultimately distort the reproducible color. In regard to our prominent contributions, adjustable bandwidths can be achieved with the resonant wavelength fixed, implying that the color saturation could be predictably controlled in a certain color domain without displacing the dominant wavelength. In an attempt to verify the performance of the proposed trans-reflective filters, the color response in transmission and reflection was evaluated with respect to the metal thickness in the International Commission on Illumination (CIE) 1931 chromaticity diagram. Finally, the color filters were assessed in terms of their angular tolerance.

## Results

### Trans-Reflective Color Filters Based on a Phase Compensated Etalon

**Figure 1:**
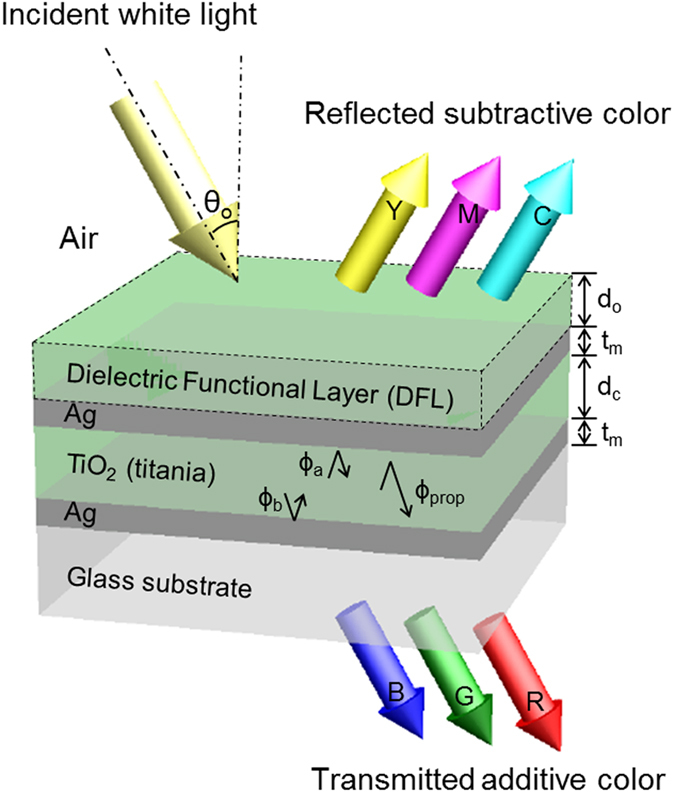
Schematic configuration of the proposed trans-reflective color filters based on a PCE, exhibiting transmissive additive tri-colors of red, green, and blue, in conjunction with reflective subtractive counterparts of cyan, magenta, and yellow.

The proposed trans-reflective color filter is based on a nano-resonator, where an MDM etalon incorporating a cavity of titania, which is sandwiched between two metallic mirrors of Ag, is integrated with a DFL of titania, as illustrated in Fig. 1. The structure is deemed to exhibit bandpass filtering characteristics in the visible band, which are represented by an intensity transmission and reflection of 
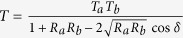
 and 
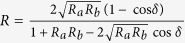
, respectively. Here, T_a_ and T_b_ are the intensity transmissivities at the top and bottom Ag-TiO_2_ interfaces, respectively, and R_a_ and R_b_ are the corresponding reflectivities^33^. The total phase accumulated during a single round-trip within the cavity is equivalent to *δ* = *ϕ*_*prop*_ _*−*_ (*ϕ*_*a*_ + *ϕ*_*b*_), where *ϕ*_*a*_ and *ϕ*_*b*_ are the reflection phase shift at the top and bottom metal-dielectric interfaces, respectively. The round-trip propagation phase shift is given by *ϕ*_*prop*_ =(4π/λ)*nd*_*c*_ for normal incidence, where n and d_c_ represent the refractive index and the cavity thickness, respectively. It is expected that for *δ* = 2*mπ* (m an integer), a transmission peak transpires at a resonant wavelength of λ_o_, which is concurrent with a reflection dip^33^.

For the trans-reflective color filters based on a PCE based nano-resonator, the cavity, metallic mirror, and DFL were designed in terms of their thickness so that the resonant spectra are obtained to render not only transmission peaks pertaining to blue (B), green (G), and red (R) but also reflection dips in relation to yellow (Y), magenta (M), and cyan (C). For the Ag and TiO_2_ materials used for the PCE, the dispersion characteristics are shown in supplementary Fig. S1. The metal thickness t_m_ was set at 25 nm. The thickness of the titania cavity (d_c_) was determined to be 52, 73, and 100 nm, corresponding to the B, G, and R bands at λ_o_ = 450, 535, and 650 nm, respectively. The DFL was chosen to be d_o_  = 50, 60, and 74 nm. The three filters, which are designated as Dev-1, Dev-2, and Dev-3, are presumed to produce primary additive colors of B, G, and R for the transmission mode, and subtractive counterparts of Y, M, and C for the reflection mode.

**Figure 2:**
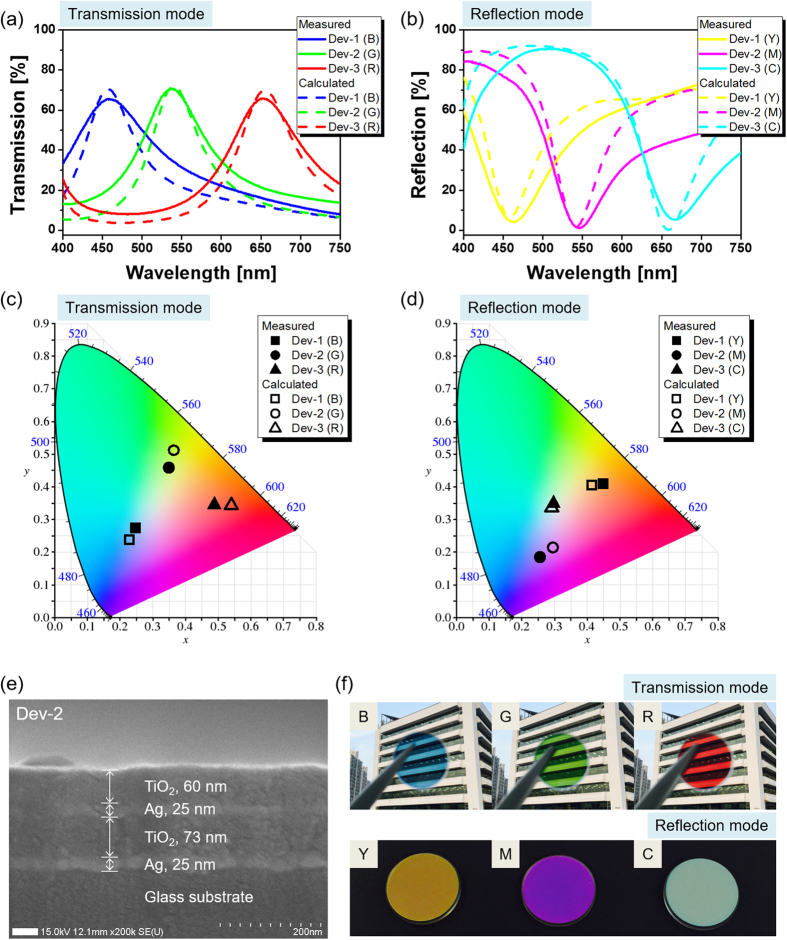
Trans-reflective color filters incorporating a phase compensated etalon. Calculated and measured spectra for (**a**) transmission and (**b**) reflection modes for Dev-1, Dev-2, and Dev-3. The corresponding color outputs are mapped in the CIE 1931 diagram for (**c**) transmission and (**d**) reflection modes. (**e**) SEM image of the fabricated Dev-2 incorporating a DFL made of titania. (**f**) Photographic images via the fabricated devices including Dev-1, Dev-2, and Dev-3, which exhibit both transmissive RGB and reflective CMY. The images were taken at Kwangwoon University by C.S. Park.

The calculated and measured spectra for the three devices are shown in Fig. 2(a,b), respectively. The color filters were manufactured by alternately depositing Ag and TiO_2_ layers on a circular glass substrate with a footprint of diameter of 25.4 mm via e-beam evaporation. Dev-1 permits a transmission window and reflection dip at a resonant wavelength of λ_o_ = 450 nm. Likewise, Dev-2 and Dev-3 exhibited similar trans-reflective filtering characteristics in the vicinity of λ_o_ = 535 and 650 nm, respectively. The colors for the transmission and reflection modes are represented in the CIE 1931 chromaticity diagram, as shown in Fig. 2(c,d), respectively. The cross-sectional scanning electron microscope (SEM) image of the fabricated color filter (Dev-2) is shown in Fig. 2(e). The Ag and TiO_2_ films belonging to the PCE are believed to have been deposited, as intended. All three devices are monitored to ensure vivid color output for the transmission and reflection modes, as shown in Fig. 2(f). Consequently, it was confirmed that our color filters achieve a satisfactory trans-reflective operation, rendering additive RGB colors in transmission and CMY colors in reflection.

### Phase Compensation Mediated by the DFL against Variations in the Thickness of the Metallic Mirrors

Considering that the output color is directly relevant to the shift in the center wavelength, we aim to explore the impact of the thickness of the metallic mirror on the transmission/reflection spectra. For the proposed color filters, the color saturation is critically dependent on the spectral bandwidth at a fixed resonant wavelength^21^. The color saturation, which is estimated from the ratio of the length from white point E (0.3333, 0.3333) to the point of interest and to the outer edge of the chromaticity diagram, signifies an attribute of visual perception, indicating the degree to which the color sensation differs from the achromatic sensation (without color) regardless of its perceived brightness^34^. The color saturation increases as the color coordinate approaches the horseshoe curve of the chromaticity diagram. In the case of a resonator color filter, the color saturation can be enhanced by increasing the thickness of the metallic mirror. However, the resonant wavelength would then be displaced as a result of variations in the reflection phase shifts in relation to the etalon structure.

**Figure 3:**
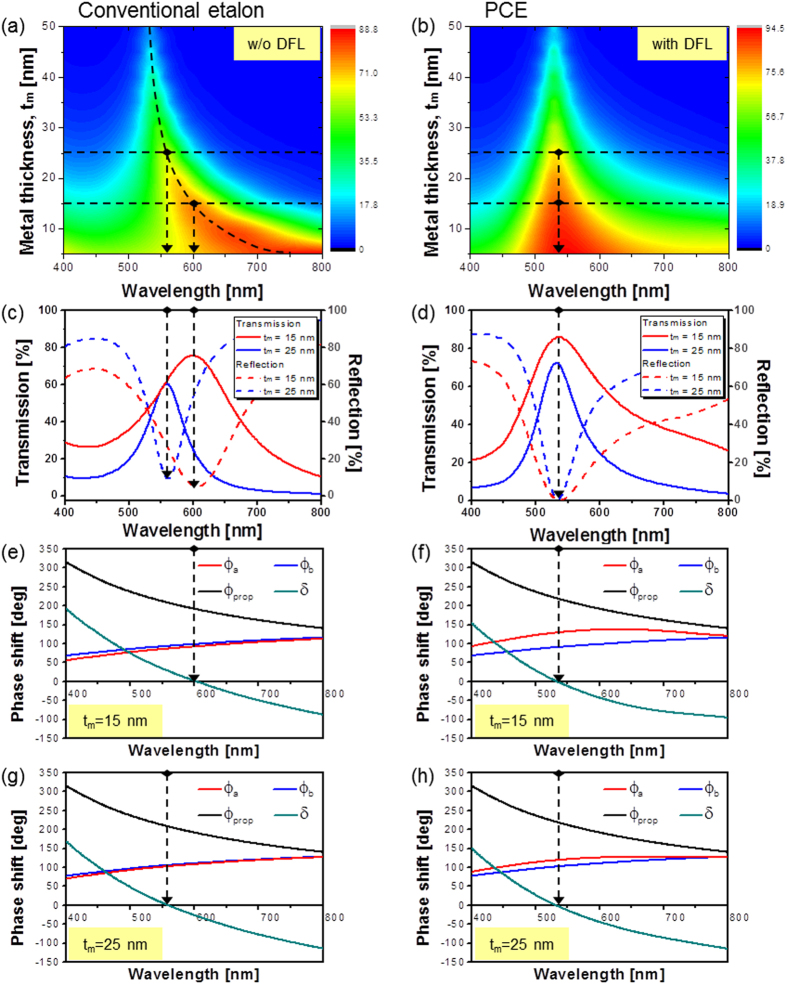
Comparison of the resonance condition for a conventional etalon and that for a PCE structure. Contour maps for the transmission spectra in response to t_m_ for (**a**) a conventional etalon and (**b**) a PCE exploiting a DFL. The transmission (solid line) and reflection (dashed line) spectra for (**c**) a conventional etalon and (**d**) a PCE for t_m_ = 15 nm and t_m_ = 25 nm, respectively. (**e**–**h**) Calculated phase shifts in nano-resonator: (**e**) a conventional etalon for t_m_ = 15 nm, (**f**) a PCE for t_m_ = 15 nm, (**g**) a conventional etalon for t_m_ = 25 nm, and (**h**) a PCE for t_m_ = 25 nm.

In order to investigate the effect of the thickness of the metallic films (t_m_) on the resonant wavelength, the transmission/reflection spectra as well as the phase shifts with t_m_ were calculated via a commercially available simulation tool, the Essential Macleod^35^. A comparative examination of the two cases of a conventional etalon and PCE incorporating the DFL was conducted in terms of the total phase, in order to validate the phase compensation mediated by the DFL. It is also noted that the DFL might serve as an anti-reflection (AR) coating to a certain extent when the thickness d_o_ is designed to mimic a quarter wavelength, satisfying *nd*_°_ =λ/4, where n is the refractive index^35^. The contour maps of the calculated transmission spectra for t_m_ ranging from 5 nm to 50 nm in steps of 5 nm for the cases of both a conventional etalon and PCE are shown in Fig. 3(a,b), respectively, with the thickness of the titania cavity fixed at 73 nm. In terms of the PCE, the DFL was 60-nm thick. Based on the contour maps for the spectra, it is shown that the bandwidth decreased with increasing metal thickness for both the conventional etalon and PCE. It is also stated that the resonant wavelength was drastically altered for the conventional etalon, whereas it remained almost constant for the proposed PCE. As for the transmission spectra according to the metal thickness, we paid attention to two specific cases of t_m_ = 15 nm and t_m_ = 25 nm both for the conventional etalon and PCE. The corresponding transmission and reflection spectra are presented in Fig. 3(c,d), respectively. The resonant wavelength is apparently invariant, irrespective of t_m_ for the PCE, whereas it is noticeably blue-shifted with increasing t_m_ for the conventional case. In order to gain better insight into the characteristic of preserving the resonant wavelength of PCE, which is imputed to the DFL, we examined the total phase pertaining to the structures, which determines the resonant wavelength λ_o_^35^. The calculated phase shift taking place within the cavity is shown in Fig. 3(e) through 3(h) for the cases of: (e) a conventional etalon with no DFL for t_m_ = 15 nm, (f) a PCE for t_m_ = 15 nm, (g) a similar conventional etalon for t_m_ = 25 nm, and (h) a PCE for t_m_ = 25 nm. The accumulated phase *δ* for the conventional etalon becomes approximately zero at λ_o_ = 600 and 555 nm, corresponding to the resonant wavelengths in the cases of t_m_ = 15 and 25 nm, respectively, as shown in Fig. 3(e,g). For the PCE with t_m_ = 15 and 25 nm, the total phase becomes nearly zero at the same λ_o_ = 535 nm, as shown in Fig. 3(f,h), respectively. The reflection phase shift relating to the top Ag-TiO_2_ interface (*ϕ*_*a*_) is precisely compensated for by the DFL made of titania, resulting in no variations in the resonance condition. It is hence verified for the PCE resonator that the resonant wavelength can be kept stable in spite of the changes in the metal thickness.

**Figure 4:**
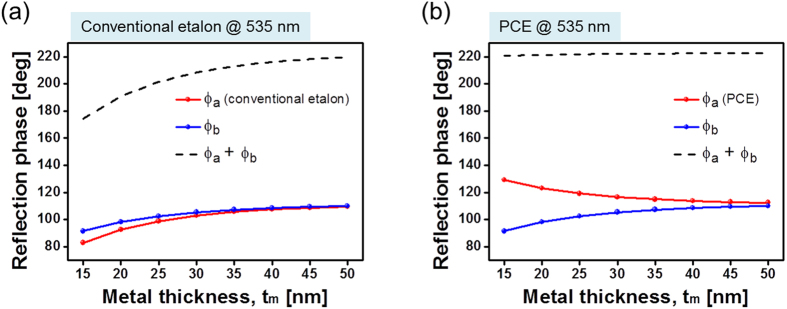
Reflection phase shift for a conventional etalon and PCE. Reflection phase shift at the top (red solid line) and bottom (blue solid line) Ag-TiO_2_ interfaces at λ_o_ = 535 nm for (**a**) a conventional etalon and (**b**) a PCE tapping into a DFL. The reflection phase, equivalent to the sum of *ϕ*_*a*_ and *ϕ*_*b*_, is plotted in terms of t_m_ as a dashed line.

For the conventional etalon and PCE, the reflection phase shifts in relation to the top and bottom Ag-TiO_2_ interfaces were observed at the resonant wavelength of λ_o_ = 535 nm, as shown in Fig. 4. The reflection phases for the thin Ag layer at the top and bottom Ag-TiO_2_ boundaries increases slightly with t_m_ for the case of the etalon with no DFL. As a result, as shown in Fig. 4(a), the reflection phase, equivalent to the sum of *ϕ*_*a*_ and *ϕ*_*b*_, drastically changes with t_m_. However, *ϕ*_*b*_ corresponding to the PCE is almost perfectly compensated for by *ϕ*_*a*_, which diminishes in the visible band, thereby maintaining a constant total reflection phase regardless of t_m_, as shown in Fig. 4(b). Since the round-trip propagation phase shift *ϕ*_*prop*_ is independent of t_m_ and the reflection phase remains constant regardless of t_m_, the resonance condition tantamount to exhibiting a near-zero phase can be fulfilled at the given wavelength, as predicted. The adoption of the DFL allows the bandwidth to be tailored without displacing the resonant wavelength. Furthermore, in a bid to achieve an etalon nano-resonator featuring adjustable bandwidths throughout the visible band, we checked the transmission and reflection spectra for the conventional etalon and PCE, when the thicknesses of the cavity were 52, 74, and 100 nm. The relevant contour maps in response to the spectra are included depending on the cavity thickness in supplementary Figs S2 and S3, where the bandwidth adjustment at a fixed wavelength is also manifested for the proposed PCE in the blue and red bands.

### Trans-Reflective Color Filters Enabling Adjustable Bandwidth at a Fixed Resonant Wavelength

**Figure 5:**
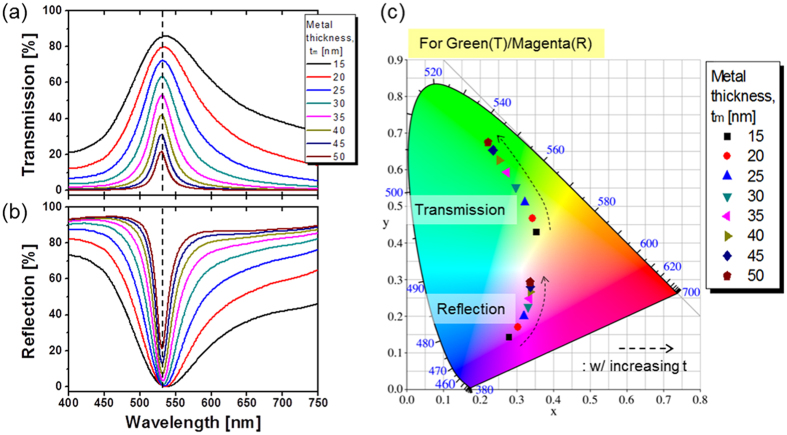
Design of trans-reflective color filter. Calculated (**a**) transmission and (**b**) reflection spectra for Dev-2, with the metal thickness varying from t_m_ = 15 nm to 50 nm in steps of 5 nm. (**c**) Corresponding CIE 1931 diagram for both transmission and reflection.

It was verified that the proposed etalon nano-resonator, incorporating a DFL, possibly enables the bandwidth to be tailored while retaining a fixed resonant wavelength λ_o_. Taking into account that a transmission peak is concurrent with the corresponding reflection dip in the visible regime, a trans-reflective color filter was created capitalizing on such a phase compensated nano-resonator. For the transmission and reflection spectra, the 3-dB bandwidth is flexibly varied by simply altering the thickness of the metallic mirrors pertaining to the resonator, and thus the color saturation for the transmitted and reflected optical output is readily controlled^21^. For a resonator filter employing a 73-nm-thick titania cavity in conjunction with a 60-nm thick DFL, the calculated transmission and reflection responses are shown in Fig. 5(a,b), respectively, when the metal thickness is changed from t_m_ = 15 nm to 50 nm in increments of 5 nm. It is found that the bandwidth becomes narrower for larger metal thickness, which is attributed to an enhanced reflectivity at both the top and bottom Ag-TiO_2_ interfaces^33^. For the filters having a 73-nm thick cavity, the performance under a trans-reflective configuration was explored by tracking the reproducible output colors in the CIE 1931 chromaticity diagram in terms of t_m_, both for the transmission and reflection modes, as depicted in Fig. 5(c). It is understood that the color saturation could be widely tuned for the PCE, entailing no significant shift in the resonant wavelength for the transmission spectra. In an effort to supplement these results, the calculated transmission/reflection spectra and the corresponding color map diagrams for the conventional etalon case are presented in supplementary Fig. S4, where a shift in λ_o_ is observed to translate into a change in the output color. It is revealed that as t_m_ increases, the color saturation is enhanced for the transmission mode responsible for additive coloration, but it is degraded for the reflection mode rendering subtractive color reproduction when t_m_ increases. In light of achieving transmissive green in conjunction with reflective magenta, the Ag layer was determined to be 25 nm thick. Two different trans-reflective color filters were additionally constructed in order to provide blue/yellow as well as red/cyan for the transmission/reflection modes, respectively. The transmission and reflection characteristics and the relevant chromaticity diagrams are shown in the vicinity of the blue and red spectral regimes, as included in supplementary Figs S5 and S6. For the three prepared trans-reflective color filters, including Dev-1, Dev-2, and Dev-3, we particularly scrutinized the influence of the thickness of the DFL on the performance in terms of the resonant wavelength and the peak transmission, as shown in supplementary Fig. S7. An appropriately designed DFL is useful not only for maintaining the resonant wavelength through the phase compensation but also for attaining the AR coating characteristic. When d_o_ deviates from the desired value equivalent to a quarter wavelength, the reflection phase at the Ag-TiO_2_ interface is altered to eventually shift the total phase pertaining to the proposed etalon filter, incurring shift in the resonant wavelength λ_o_. Consequently, the DFL with a thickness of quarter wavelength plays a dual-role of preserving λ_o_ and suppressing reflection as an AR coating.

**Figure 6:**
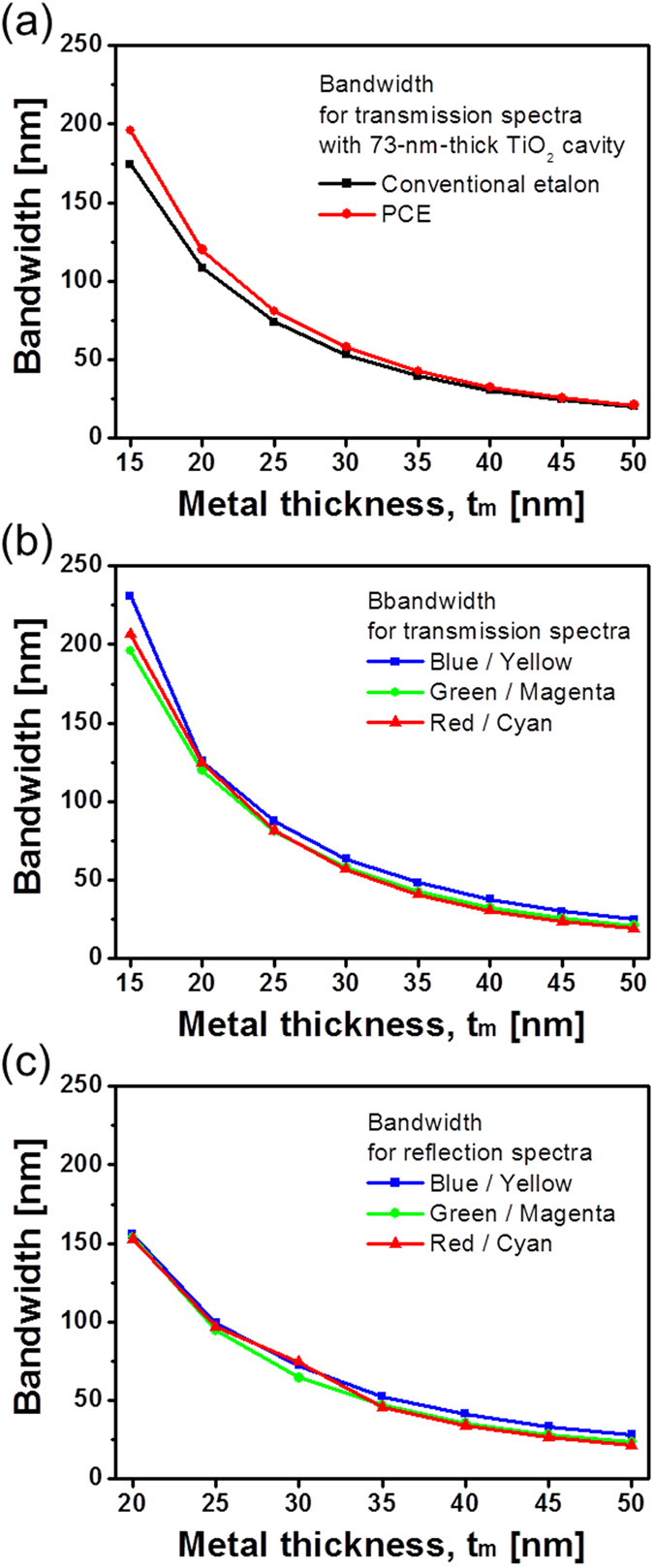
Comparison of spectral bandwidths. (**a**) Calculated bandwidths for the transmission spectra between the conventional etalon and PCE. Calculated bandwidths for (**b**) transmission and (**c**) reflection spectra for three different cases of PCE, which draw upon titania cavities of d_c_ = 52, 73, and 100 nm.

With the aim of inspecting the bandwidth tailoring for the resonator filters, which have a 73-nm-thick cavity made of titania, the transmission bandwidth hinging on the presence of the DFL is plotted in Fig. 6(a). Figure 6(b,c) show the transmission and the reflection bandwidths for the three PCE structures, operating in the B, G, and R bands. It was observed that the transmission and reflection bandwidths decrease with increasing t_m_ both for the conventional and the PCE structures. For the three PCE filters having titania cavities of d_c_ = 52, 73, and 100 nm, the transmission and reflection bandwidths were approximately 83 and 97 nm for t_m_ = 25 nm, respectively. This demonstrates that the color saturation can be primarily enhanced by narrowing the bandwidth of the passband of the filter for a reduced sideband level^21^. In this work, we attempted to vary the bandwidth by altering the thickness of the metallic mirror films, which determines the reflectivity of the top and bottom Ag-TiO_2_ interfaces, thereby diminishing both the bandwidth of the filter as well as its sideband level.

**Figure 7:**
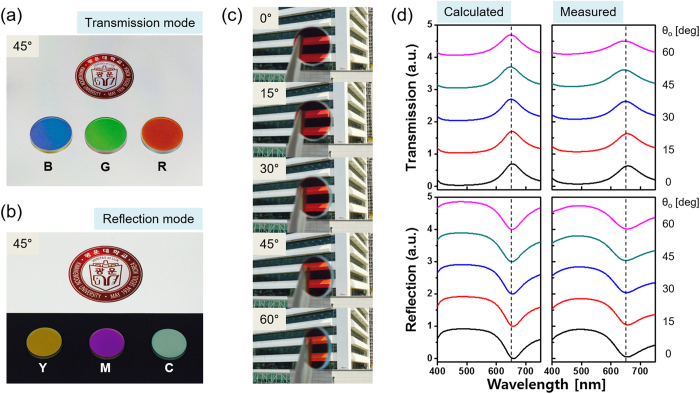
Angle independent properties of the trans-reflective color filters. Captured output images for an oblique incidence of θ_o_ = 45° exhibiting (**a**) transmissive RGB and (**b**) reflective CMY. (**c**) Photographic images via the fabricated Dev-3, which were taken at Kwangwoon University by C.S. Park, and (**d**) (left) Calculated and (right) measured transmission and reflection spectra, for different angles of incidence varying from θ_o_ = 0 to 60° at an interval of 15°.

Based on the bandwidth adjustment and color map diagrams, we created three devices, including Dev-1, Dev-2, and Dev-3. Each device is deemed to deliver primary additive colors of B, G, and R for the transmission mode while producing subtractive counterparts of Y, M, and C for the reflection mode. It should be noted that the subtractive color should offer twice the photon throughput of its additive counterpart, which ultimately translates into elevated efficiencies and strengthened color signals^9^,^10^. From the viewpoint of their applications as a beam splitter, the proposed trans-reflective color filters are strongly preferred for accomplishing an angle tolerant performance. In order to support the angle insensitive performance of the fabricated color filters, we have monitored their color outputs by altering the angle of incidence from θ_o_ = 0 to 60°. Figure 7(a,b) first display the output color images corresponding to the RGB of the transmission mode and the CMY of the reflection mode, respectively, for θ_o_ = 45°. As shown in Fig. 7(c), a group of pictures were also taken of a building via the red color filter, Dev-3, by varying the angle from θ_o_ = 0 to 60° at an interval of 15°. The transmission and reflection spectra for the device were then measured using a spectrophotometer (PHOTON RT, Essent Optics Ltd.), as plotted in Fig. 7(d), by similarly varying the incident angle. Based on the results given in Fig. 7(a) through 7(d), it was found that the proposed red color filter resulted in no significant angle dependent variations in terms of the produced color and the spectral response in the transmission and reflection modes. The rest of the filter devices were similarly verified to give rise to equivalent angle insensitive performance. For Dev-1 and Dev-2, their color outputs and transmission/reflection spectra with the incident angles are particularly provided in supplementary Fig. S8. Due to their conspicuous properties such as angle insensitive color reproduction, the proposed PCE based color filters are anticipated to play a vital role as a beam splitting device in applications, spanning the microscopy, beam projectors, and 3D hologram.

## Discussion

In order to develop trans-reflective color filters, we present a phase compensated nano-resonator resorting to a functional layer, enabling bandwidth adjustment at a fixed resonant wavelength. The nano-resonator consists of a Ag-TiO_2_-Ag etalon structure overlaid with a thin titania film serving as a DFL, thus forming a (Ag-TiO_2_-Ag)|TiO_2_ configuration. The resonance condition was theoretically investigated in terms of the phase shift depending on the thickness of the Ag metallic mirror, so that the feasibility of the phase compensation empowered by the DFL could be validated. It is shown that the DFL helps provide a conspicuous feature of bandwidth adjustability at a fixed resonant wavelength irrespective of the metal thickness, allowing for flexible control of the color saturation. This is supported by the corresponding color response evaluated in the chromaticity diagram, whereby the proposed PCE permitting adjustable bandwidths could exhibit a wide range of color saturation in both transmission and reflection. The manufactured devices could demonstrate a peak reflection of ~90% and a peak transmission of ~70%, in conjunction with well-defined bandpass/bandstop characteristics, comparable to the performance of the colorant dye-based filters^36^,^37^. It is also verified via simulations that the spectral performance of the proposed color filters can be further enhanced by optimizing the thickness and optical properties of the metallic and dielectric layers. Such filters are predicted to be applied to embody color pixels for a variety of display and imaging devices. In addition, while the conventional multilayered color filters, used for color display or imaging applications, are typically comprised of as many as 20 layers, the proposed device involves much fewer metal-dielectric stacks so as to reproduce vivid color output. Taking into account such previous works as reported in ref. 38, which are based on a similar stacked film structure to the proposed scheme, we anticipate that our devices can be readily implemented to be suitable for colored imaging applications requiring a pixel resolution. Moreover, it should be noted the proposed color filters enable an angle insensitive performance, unlike the conventional multilayer based approaches. It is hence anticipated that the proposed filters can play a central role in various applications encompassing the holography, fluorescence microscopy, CCD imaging, etc.

## Methods

### Simulation

Calculations for obtaining transmission/reflection spectra and phase shifts were conducted using Essential Macleod (Version 9.8.436), which is a commercially available tool specialized for the analysis of thin-film structures.

### Device fabrication

The proposed color filters were manufactured on a disc substrate made of polished B270 glass, with a diameter of 25.4 mm. Prior to the deposition of thin films, organic and inorganic contaminants on the substrate were removed via successive ultrasonification in acetone, ethanol, and deionized water. A bottom Ag film of 25-nm thickness, a TiO_2_ cavity, which has different thicknesses of 52, 73, and 100 nm, a top Ag film of 25-nm thickness, and a TiO_2_ DFL with thicknesses of 50, 60, and 74 nm, were subsequently deposited for the three devices of Dev-1, Dev-2, and Dev-3, respectively. The film deposition was fulfilled via e-beam evaporation on the glass substrate.

### Optical characterization

A cross-sectional structure of the prepared Dev-2 was observed under a high-resolution scanning electron microscope (Hitachi Ultra-high Resolution SEM S-4800), as shown in Fig. 2(e). For the grown films, the thickness and index of refraction were verified using a reflecto-spectrometer (Filmtek4000, SCI), operating in the spectral range of 450 to 1,650 nm. The transmission and reflection spectra were measured in terms of the incident angle using spectrophotometers (Varian Cary 500, Varian, Inc.) and (PHOTON RT, Essent Optics Ltd).

## Additional Information

**How to cite this article**: Park, C.-S. *et al.* Trans-Reflective Color Filters Based on a Phase Compensated Etalon Enabling Adjustable Color Saturation. *Sci. Rep.* **6**, 25496; doi: 10.1038/srep25496 (2016).

## Supplementary Material

Supplementary Information
