# Supplementary material for: Trans-Reflective Color Filters Based on a Phase Compensated Etalon Enabling Adjustable Color Saturation
Source: Sci Rep. 2016 May 6;6:25496. doi: 10.1038/srep25496 (PMC4858682; doi:10.1038/srep25496)
Supplement: Supplementary Information [file srep25496-s1.doc]

Supplementary Information

**Trans-Reflective Color Filters Based on a Phase Compensated Etalon Enabling Adjustable Color Saturation**

*Chul-Soon Park^1^, Vivek Raj Shrestha^1^, Sang-Shin Lee^1^,^*^ and Duk-Young Choi^2^*

^1^Department of Electronic Engineering, Kwangwoon University,

20 Kwangwoon-ro, Nowon-gu, Seoul 01897, South Korea

^2^Laser Physics Centre, Research School of Physics and Engineering,
Australian National University, Canberra, ACT 2601, Australia

^*^E-mail: *slee@kw.ac.kr*

The dispersion characteristics of silver (Ag) and titania (TiO_2_), used for the design and analysis of the nano-resonator based color filters, are plotted in Figure S1. Data for TiO_2_ were obtained from the ellipsometric measurements for a deposited film while data for Ag were obtained from Palik [1].


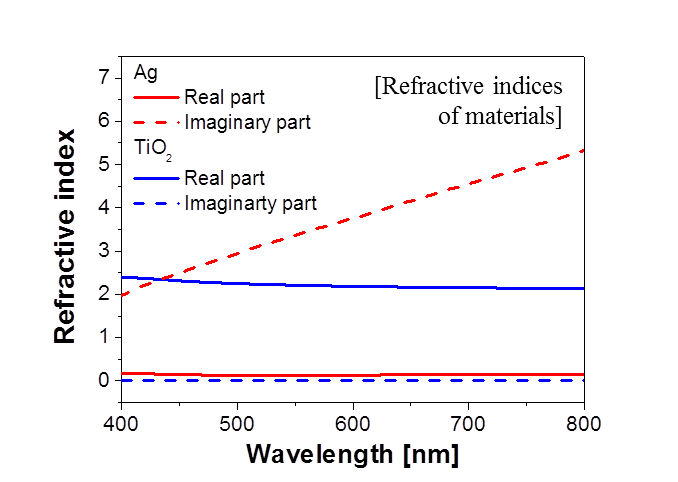


**Figure S1.** Refractive indices of Ag and TiO_2_ used for the calculation.

Figures S2 and S3 show the contour maps for the transmission and reflection spectra with respect to the metal thickness t_m_ for the conventional etalon and the phase compensated etalon (PCE), respectively.

**
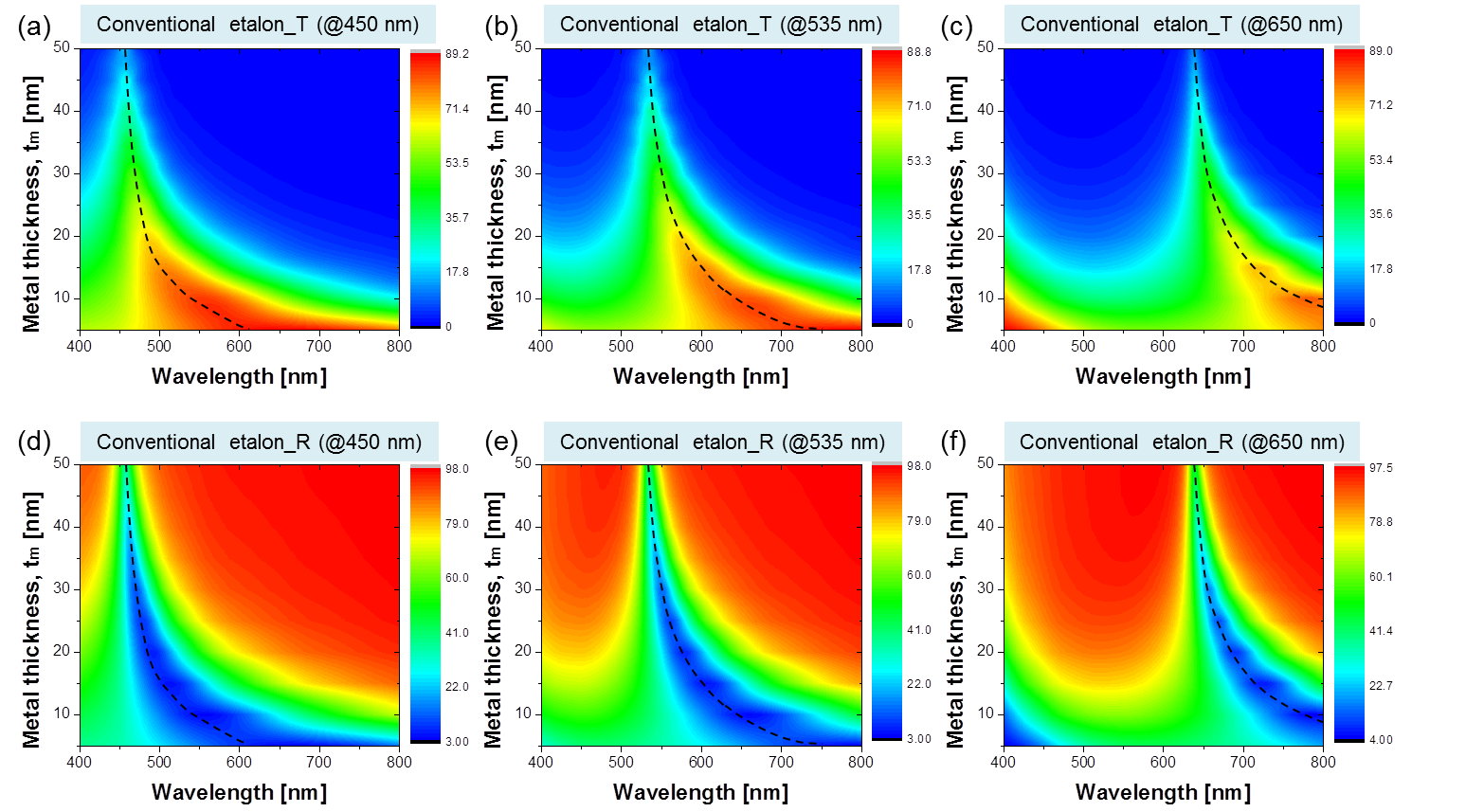
**

**Figure S2.** Contour maps of (a-c) transmission and (d-f) reflection spectra with different metal thicknesses, t_m_ for conventional etalon structures.

**
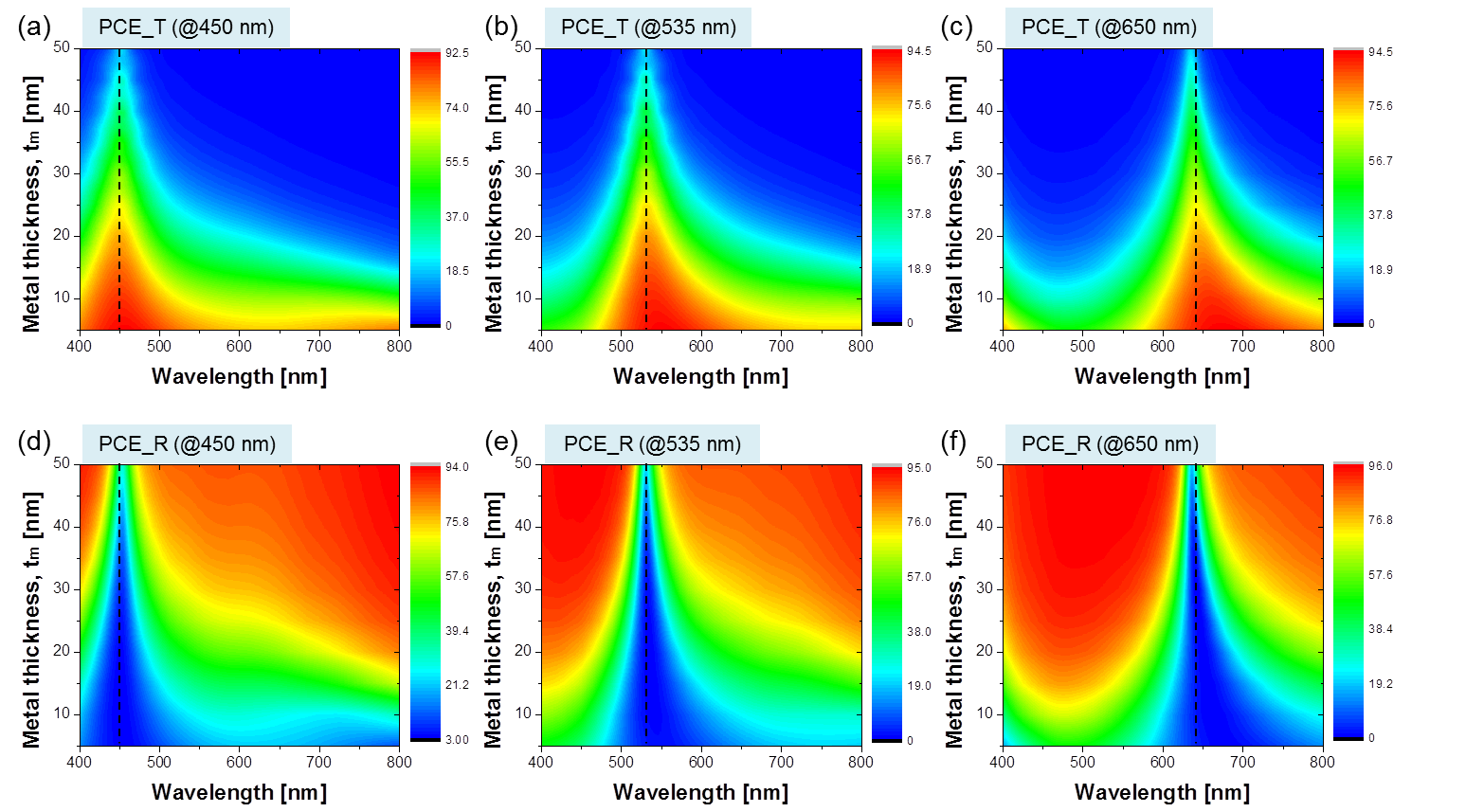
**

**Figure S3.** Contour maps of (a-c) transmission and (d-f) reflection spectra with different metal thicknesses, t_m_ for PCE structures.

Figure S4 shows the calculated transmission and reflection spectra, and the corresponding CIE 1931 color map diagram for the conventional etalon with a 73-nm-thick cavity. Shift in λ_o_ is observed to result in a remarkable difference in the represented colors.

**
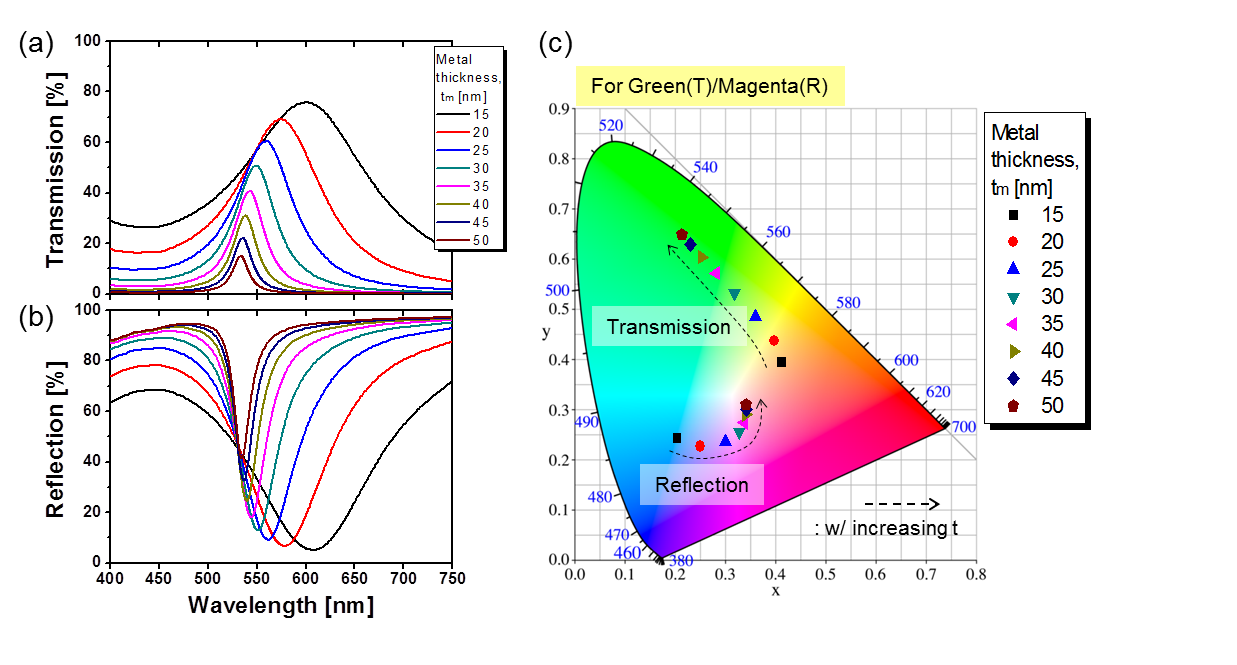
**

**Figure S4.** (a) Calculated transmission and (b) reflection spectra for a conventional etalon with a cavity of 73-nm thickness, with t_m_ ranging from 15 nm to 50 nm. (c) Corresponding CIE 1931 diagram for both transmission and reflection.

Figures S5 and S6 show the transmission and reflection spectra for Dev-1 and Dev-3, respectively, with respect to t_m_ varying from 15 nm to 50 nm in steps of 5 nm. The corresponding CIE 1931 chromaticity diagrams are also represented.


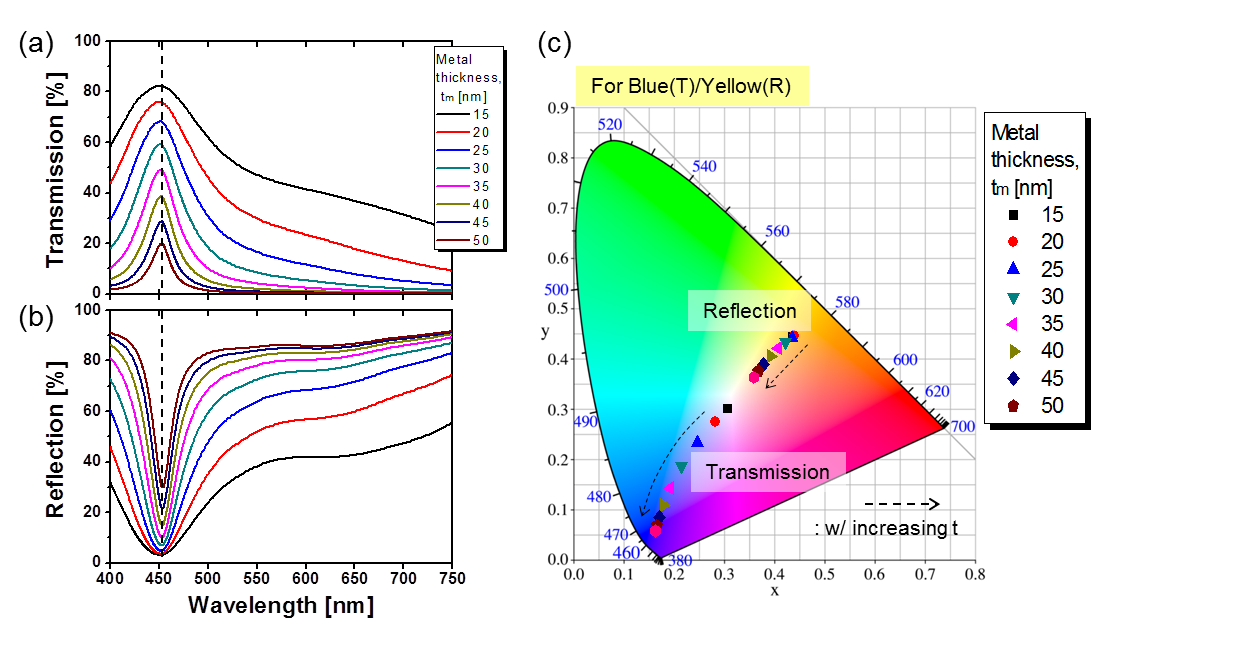


**Figure S5.** (a) Calculated transmission and (b) reflection spectra for Dev-1, with t_m_ ranging from 15 nm to 50 nm. (c) Corresponding CIE 1931 diagram for both transmission and reflection.


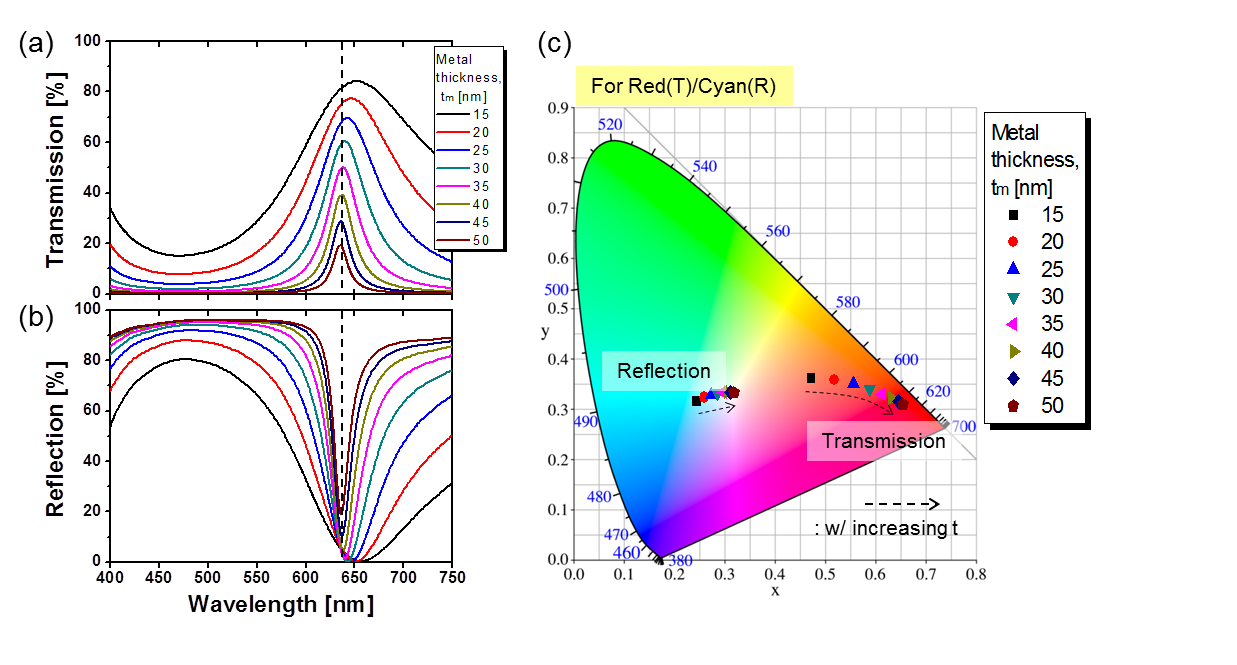


**Figure S6.** (a) Calculated transmission and (b) reflection spectra for Dev-3, with t_m_ ranging from 15 nm to 50 nm. (c) Corresponding CIE 1931 diagram for both transmission and reflection.

Figure S7 describes the influence of the thickness d_o_ of the dielectric functional layer (DFL) on the performance of the color filters in terms of the resonant wavelength and peak transmission.

**
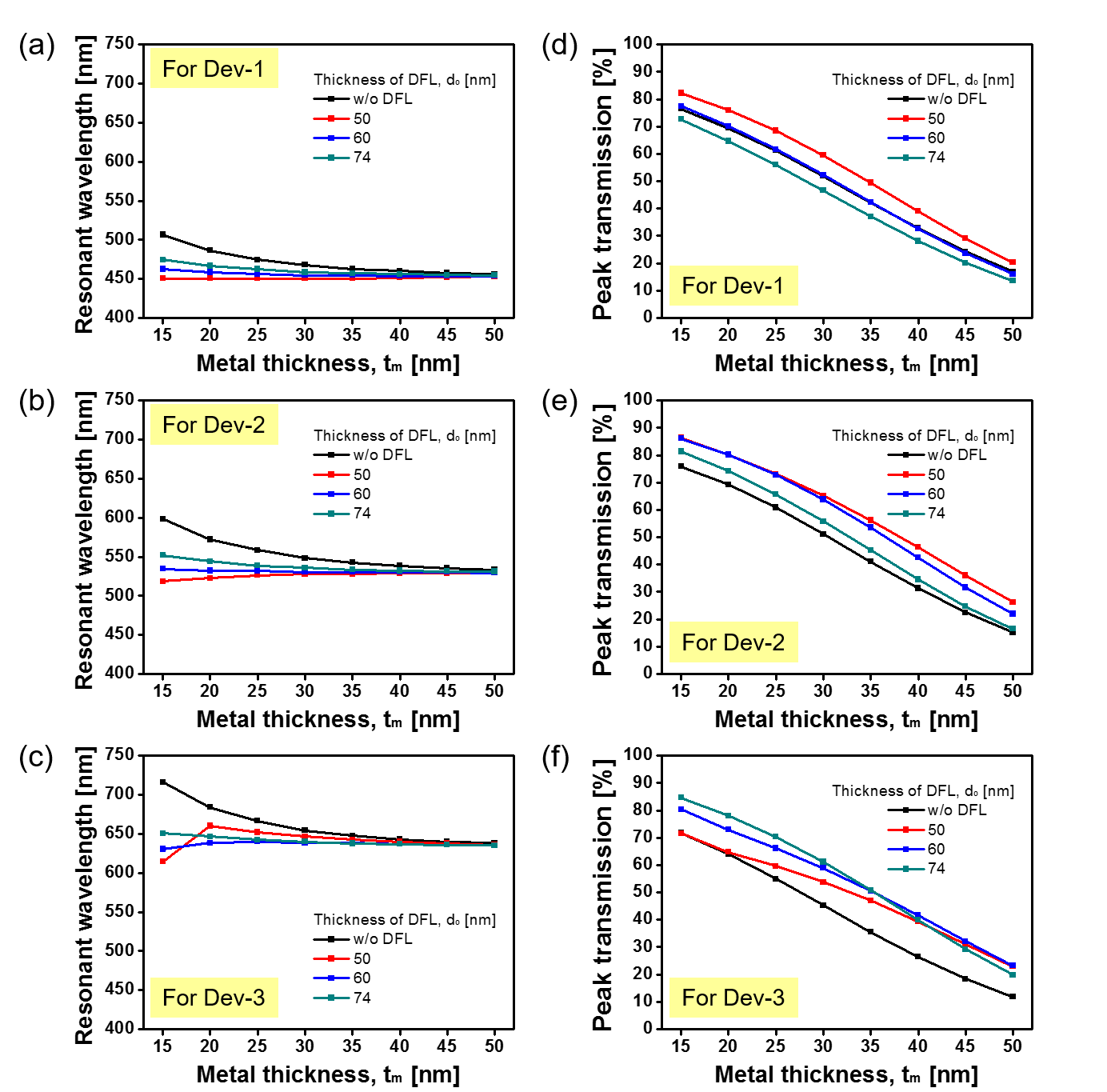
**

**Figure S7.** (a-c) Calculated resonant wavelength and (d-f) peak transmission for different metal thicknesses t_m_ in terms of the thickness d_o_ of the DFL for Dev-1, Dev-2, and Dev-3.

Figure S8 shows the color outputs and transmission/reflection spectra for Dev-1 and Dev-2 for incident angles ranging from θ_o_ = 0 to 60° at an interval of 15°, respectively


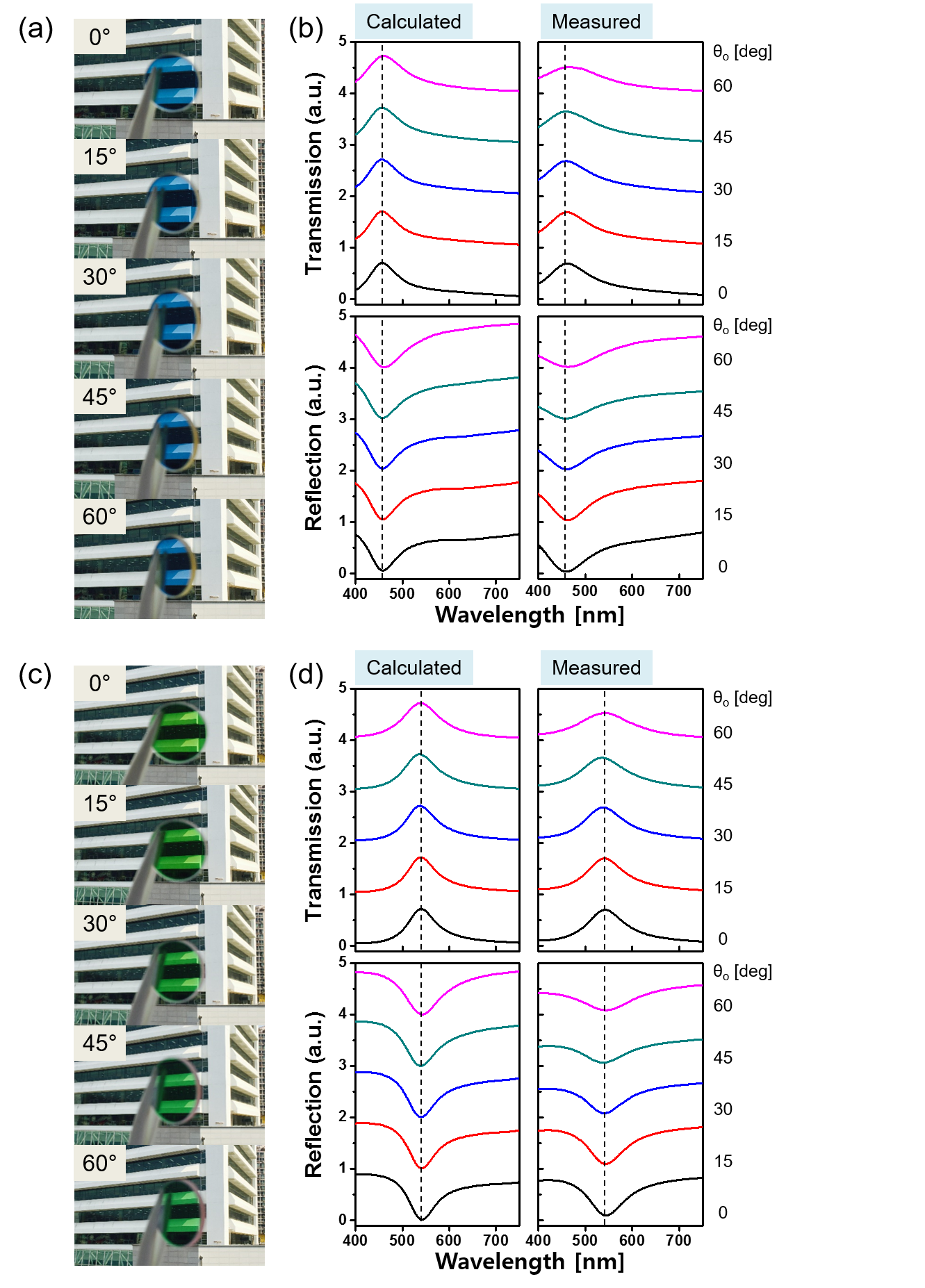


**Figure S8.** (a) Photographic images at different angles of incidence and (b) (left) calculated and (right) measured transmission and reflection spectra for Dev-1. (c) Similar photographic images and (d) (left) calculated and (right) measured transmission and reflection spectra for Dev-2.

**References**

[1] E. D. Palik, *“Handbook of Optical Constants of Solids III,”* (Academic Press, San Diego, USA, 1998).
